# Improving Access to Medicines in Poor Countries: The Role of Universities

**DOI:** 10.1371/journal.pmed.0030136

**Published:** 2006-04-18

**Authors:** Dave A Chokshi

## Abstract

Universities Allied for Essential Medicines, a coalition of students and faculty across North America, focuses on how academic research institutions can help to improve access to essential medicines.

According to the World Health Organization, about 10 million people—most of them in low- and middle-income countries—die needlessly every year because they do not have access to existing medicines and vaccines [1]. Countless others suffer from neglected tropical diseases, such as sleeping sickness, lymphatic filariasis, and blinding trachoma, for which there are still too few safe or effective medicines [2]. Drug companies have traditionally been reluctant to develop drugs for neglected diseases because the patients are too poor to pay for them, so there is no financial incentive for drug development.

Comprehensive solutions are thus needed to increase both access to existing medicines and research on neglected diseases. These solutions must involve strengthening health-care systems in poor countries, increasing financial flows for the most pressing public health crises, and better matching our research and development efforts to the needs of the poor. The challenges of making such wholesale changes are daunting [3]. Our organization, Universities Allied for Essential Medicines (UAEM) (Box 1), a coalition of students and faculty at about 25 universities across North America, focuses on the role of academic research institutions as a starting point for closing the access and research gaps. To be sure, much of the recent progress in global health research and awareness can be attributed to universities. Yet we must go further. As medical students, we have a unique ability to “turn the spotlight inward” by calling attention to our universities' responsibilities when it comes to essential medicines.

## Why Universities?

University research is vital to the development of new medicines; total biomedical research expenditures at universities were US$19.6 billion in 2002 [4,5]. Meanwhile, the principles that guide universities—creating and disseminating knowledge for public benefit—are well aligned with the goal of improving access to medicines globally. In addition, many universities have offices responsible for transferring academic innovations (those arising from university research) to the commercial sector. Such transfer allows these innovations to be further developed and marketed so that they can benefit the public; universities rarely have the resources to do such development and marketing themselves. University technology transfer offices aim to embody the same guiding principles as those of the university itself. For example, the Center for Technology Transfer at the University of Pennsylvania explicitly states that its chief objective is to “commercialize Penn research discoveries for the public good” (see http://www.ctt.upenn.edu/).

University research is vital to the development of new medicines.

In most instances, the transfer of an innovation from a university to a for-profit company means that the university relinquishes control over the subsequent development and marketing of a medicine. This raises the possibility that the company will put the medicine out of reach of poor patients, either by charging prices that poor patients cannot afford or through legal maneuvers that otherwise restrict access in poor countries. However, two recent cases demonstrate that universities can influence access to such medicines.

First, in 2001, the humanitarian organization Médecins Sans Frontières sought the permission of Yale University to use a generic version of Zerit (stavudine), an antiretroviral drug for HIV infection, to treat South African patients. Médecins Sans Frontières made this request because it had gathered evidence that generic stavudine could be purchased for a fraction of the cost of the expensive branded version available in South Africa; the cost savings would permit an expansion in the number of patients who could be treated for HIV. Yale University owned the patent for stavudine, but the university had granted an exclusive license that conferred intellectual property rights for the medicine to the drug company Bristol-Myers Squibb (New York, New York, United States of America). The request from Médecins Sans Frontières prompted global attention and intense discussions between the university and Bristol-Myers Squibb [6]. The result was the first patent concession on an HIV drug—that is, Bristol-Myers Squibb allowed generic stavudine to be bought and sold within South Africa—and a 30-fold reduction in the price of the patented drug in South Africa. The impact of this intervention from Médecins Sans Frontières, and from Yale's negotiations with the drug company, was indisputable. Rapid expansion of HIV-treatment programs in sub-Saharan Africa would not have been possible without the widespread availability of generic stavudine, a treatment recommended by the World Health Organization as first-line therapy for HIV/AIDS [7].

A second example further demonstrates the leverage universities can have in improving access to medicines. Scientists at Emory University (Atlanta, Georgia, United States of America) had conducted research that contributed to the development of the antiretrovirals, Emtriva (emtricitabine) and Truvada (emtricitabine and tenofovir). These discoveries were transferred to industry through an intellectual property agreement that stipulated that the university would receive royalty payments for any drugs developed from the Emory research. Last year, in a deal with Gilead Sciences (Foster City, California, United States of America) and Royalty Pharma (New York City, New York, United States of America), Emory sold its rights to those royalties for a lump sum payment of US$525 million [8].

The magnitude of the deal, which was the largest-ever transaction of its kind for an academic institution, caught the attention of student activists at Emory, who investigated Gilead's provisions for access to Emtriva and Truvada in poor countries and found them lacking [9]. Emory students are currently engaged in discussions with the university administration about Gilead's access practices, armed with a straightforward but cogent argument: Emory could have received the same royalty payment while advocating for greater access to Emtriva and Truvada for patients in poor countries. That is, expanding access does not require that universities sacrifice their bottom line. The reason for this is simple: the patients who aren't currently able to afford the drugs they so desperately need do not factor into either Gilead's revenue or (by extension) Emory's royalties.

## Closing the Access and Research Gaps: Policy Proposals for Universities

The case of Emory and the two medicines Emtriva and Truvada highlights the difficulty of crafting retrospective solutions to problems that should have been foreseen. Ideas on how to prevent similar situations from arising in the future have been circulating in academic and policy circles over the past two years. For instance, in 2005 the American Academy of Arts and Sciences (Cambridge, Massachusetts, United States of America) published a report exploring how to license university discoveries to drug companies in a way that still ensures that the drugs can be accessed for humanitarian uses [10]. The report argued that humanitarian licensing practices would involve “a provision in a license whereby inventors and technology suppliers protect in advance the possibility of sharing their proprietary technology with third parties for the benefit of people in need.” The Association of University Technology Managers (Northbrook, Illinois, United States of America) has convened a group known as Technology Managers for Global Health to look at how university research can be optimally exploited to advance global health outcomes (http://www.tmgh.org). Our own organization, UAEM, has drafted recommendations that we advocate for individual institutions through our university-based chapters [11].

UAEM proposes that universities make changes in both their principles and policies in order to improve access to medicines in poor countries. We recommend that universities adopt an official resolution that improving global human welfare is the most important goal of university technology transfer. To satisfy this principle, we put forward two specific policy proposals: (1) universities should adopt licensing provisions that facilitate access to their health-related innovations in poor countries, and (2) universities should promote research on neglected tropical diseases and find ways to work with nontraditional partners (such as developing-world research institutions and public–private partnerships) that seek to develop medicines for these diseases.

We advocate a set of humanitarian licensing provisions known as “equitable access licensing,” which is designed to do a number of things that traditional university licenses typically do not do. For example, under the Equitable Access License (EAL), when certain conditions in the license are met (e.g., when a generic pharmaceutical company in a poor country notifies the university that a key medicine is overpriced there), patent barriers are lifted. Under the EAL, the intellectual property required to make that product is open to anyone that wants to use it to increase access in poor countries. And so a generic pharmaceutical company wanting to produce a medicine in a poor country won't get sued for doing so, as long as the conditions that trigger the license are met.

Beyond humanitarian licensing, we advocate the institution of policies to promote neglected-disease research. Specifically, we recommend that the universities facilitate participation in innovative research activities such as public–private partnerships (in which the public sector teams up with the commercial sector). We also recommend that universities promote projects that hold potential for neglected-disease drug development [12]. Such promotion includes removing any barriers that prevent university scientists from accepting research funding from public–private partnerships, proactively monitoring university innovations for potential neglected-disease applicability, and ensuring that university intellectual property does not serve as an impediment for scientists working on neglected diseases, either within universities or elsewhere. Full details of both the EAL and our neglected-disease policies have been laid out elsewhere [13].

## Addressing Counterarguments

The unique appeal of an EAL is that it promotes true generic competition in poor countries. We anticipate, however, that the feasibility of our proposal will raise a number of doubts, some of which we attempt to address here.

First, it is important to note that for any given product, a pharmaceutical company's bottom line would remain relatively intact. Equitable Access Licensing works by dividing the world pharmaceutical market between rich and poor countries. Consider, for example, any university innovation that has been developed into a drug. That drug can remain under patent protection in high-income countries, where the pharmaceutical industry earns the vast majority of its revenue. Generic competition is allowed only in markets where there is little access—and, therefore, little revenue—in the first place. [Fig pmed-0030136-g001]

**Figure pmed-0030136-g001:**
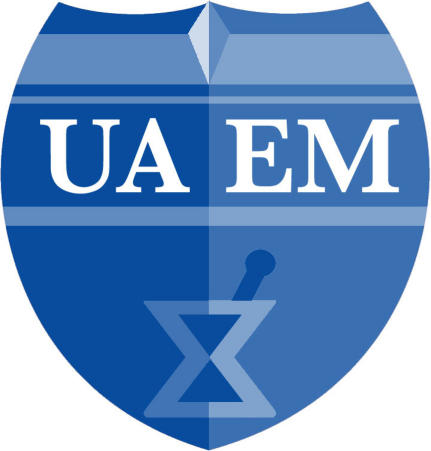
Logo of Universities Allied for Essential Medicines (Image: UAEM)

Licensees may express disquiet about cheaper generic products overcoming regulatory (customs) barriers and entering high-income markets illegally. However, there is no empirical evidence of any substantial flows of medicine from poorer countries to high-income countries [14]. Insofar as such diversion is a concern, EAL signatories can address it in the same manner that the World Trade Organization has—by requiring the use of different packaging, pill color, and pill shape in different countries to facilitate the identification of illegal imports [15].

Another concern universities may have is whether the EAL is financially viable for universities. This concern is not justified, because pharmaceutical companies would not lose a significant amount of revenue as a result of the EAL, and any decrease in licensing revenue at a given university would be vanishingly small. The fact that licensing revenues typically account for about 4% of university research funds underscores the point that universities would not suffer ill effects from implementing Equitable Access Licensing [16].

Finally, aside from any intangible benefits research institutions might derive from being leaders in responding to an important humanitarian issue, there are reasons to believe that pioneering universities stand to gain financially by adopting our proposals. Combining accessoriented licensing policies with an augmented neglected-disease research agenda can help universities position themselves as research centers for foundation-sponsored partnerships. The burgeoning field of public–private partnerships for global health research has attracted over US$1.2 billion in funding from sources such as the Gates Foundation, the vast majority of which is contracted out to research scientists [17]. The University of California Berkeley (Berkeley, California, United States of America) has recently begun marketing its “Socially Responsible Licensing Initiative” as a way to attract some of this nontraditional funding and has already signed a handful of deals with foundations and other nonprofits under that licensing rubric [18]. In our role as students, UAEM members have even loftier aspirations: to foment a collective movement that ensures that our universities' innovations reach those who need them the most.

**Box 1. Universities Allied for Essential Medicines**

**Who We Are**

- UAEM is a coalition of students and faculty at about 25 research universities across North America.
- Our goal is to improve access to medicines in poor countries through university action.

**What We Do**

- Our activities take place at both the chapter level and the international level.
- At the chapter level, we petition for changes in the policies and practices at the universities we attend. For example, at the University of California Berkeley, administrators announced a Socially Responsible Licensing Initiative (http://ipira.berkeley.edu/docs/sociallyresponsible.pdf) that arose in part through discussions with the Berkeley UAEM chapter.
- At the international level, we convene groups of students—in consultation with faculty members and other experts—to determine how best to improve access to medicines in poor countries through research and policy analysis. For example, a consensus UAEM Policy Statement was released in October 2005 after a meeting at Georgetown University (Washington, D. C., United States of America) that brought together more than 75 students representing 28 universities (see http://www.essentialmedicine.org/Oct2005PolicyStatement.pdf).

**How You Can Get Involved**

- Join UAEM through our Web site (http://www.essentialmedicine.org).
- Figure out what steps your university currently takes to ensure access to its innovations in poor countries by talking to faculty members, technology transfer officers, and administrators who set the university research agenda.
- Learn more about the access and research gaps through organizations such as Médecins Sans Frontières (http://www.accessmed-msf.org) and build awareness on your own campus.

## Abbreviations

EAL: Equitable Access License
UAEM: Universities Allied for Essential Medicines
